# Use of 18F-FDG-PET/CT in differential diagnosis of primary central nervous system lymphoma and high-grade gliomas: A meta-analysis

**DOI:** 10.3389/fneur.2022.935459

**Published:** 2022-08-17

**Authors:** Guisheng Zhang, Jiuhong Li, Xuhui Hui

**Affiliations:** ^1^Department of Neurosurgery of West China Hospital, Sichuan University, Chengdu, China; ^2^West China School of Medicine, West China Hospital, Sichuan University, Chengdu, China; ^3^Department of Neurosurgery, Minda Hospital of Hubei Minzu University, Enshi, China

**Keywords:** primary central nervous system lymphoma, high-grade gliomas, PET/CT, diagnosis, meta-analysis

## Abstract

**Background:**

Primary central nervous system lymphoma (PCNSL) and high-grade glioma (HGG) appear similar under imaging. However, since the two tumors vary in their treatment methods, their differential diagnosis is crucial. The use of 18F-fluorodeoxyglucose positron emission tomography computed tomography (18F-FDG-PET/CT) imaging to effectively distinguish between the two tumors is not clear; therefore, a meta-analysis was carried out to determine its effectiveness.

**Materials and methods:**

The databases PubMed, EMBASE, Cochrane, Web of Science, China National Knowledge Infrastructure (CNKI), Wanfang, China Science, and Technology Journal Database (CQVIP) were exhaustively searched using stringent inclusion and exclusion criteria to select high-quality literature. The Quality Assessment Tool for Diagnostic Accuracy Studies (QUADAS-2) was used for the qualitative assessment of the included literature. The bivariate effect model was used to combine statistics such as sensitivity (SEN) and specificity (SPE), positive likelihood ratio (PLR), negative likelihood ratio (NLR), and diagnostic odds ratio (DOR) [95% confidence intervals (CI)], plot summary receiver operating characteristic (SROC) curve, and calculate the area under the curve (AUC) value. Sensitivity analysis was used to evaluate the stability of the results, and Deek's test was used to assess publication bias. Meta-regression and subgroup analysis was used to determine the sources of heterogeneity.

**Results:**

A total of nine studies were included in this study. For differential diagnosis of PCNSL and HGG, the combined SEN was 0.91 (95% CI: 0.80–0.96; I^2^ = 46.73%), combined SPE was 0.88 (95% CI: 0.82–0.93; I^2^ = 56.30%), the combined PLR was 7.83 (95% CI: 4.96–12.37; I^2^ = 15.57%), combined NLR was 0.10 (95% CI: 0.05–0.23; I^2^ = 31.99%), combined DOR was 77.36 (95% CI: 32.74–182.77; I^2^ = 70.70%). The AUC of SROC was 0.95 (95% CI: 0.93–0.97). No publication bias was found and the sample size and different parameters were the primary reason for heterogeneity.

**Conclusion:**

The 18F-FDG-PET/CT imaging technique has a high diagnostic accuracy in the differential diagnosis of PNCSL and HGG. Patients suspected to have the above two tumors are suggested to be examined by 18F-FDG-PET / CT to help in the clinical distinction and further treatment modalities.

## Introduction

Primary central nervous system lymphoma (PCNSL) is a rare, extra nodal non-Hodgkin's lymphoma, which accounts for nearly 2% of all primary brain tumors (1). The most common pathological type of PCNSL is diffuse large B-cell lymphoma, which is likely to occur in immunodeficient patients (2, 3). Conventionally, chemotherapy and / or radiotherapy are the preferred treatment modalities for PCNSL, with surgery being considered in rare cases (3). Glioma refers to the tumor that originates from glial cells of the brain and is a highly prevalent primary intracranial tumor (2). According to the 2021 WHO classification of central nervous system tumors, glioma is divided into grade 1 to 4: grade 1 and 2 are low-grade gliomas (LGG), while grade 3 and 4 are high-grade gliomas (HGG). Surgical treatment with postoperative selective adjuvant chemotherapy or radiotherapy is the primary choice of treatment for patients with LGG (4). HGG also usually requires surgical treatment together with postoperative adjuvant chemotherapy or radiotherapy (5). Owing to the significant differences between the treatment methods for the two tumor patients, it is crucial and clinically significant to accurately distinguish PCNSL and HGG.

Even today, the gold standard for the diagnosis of PCNSL and HGG is histopathology. Non-invasive imaging evaluation prior to treatment is highly valuable for clinical treatment and diagnosis. The conventional Magnetic Resonance Imaging (MRI) technology, relying on morphology, is the most commonly used imaging tool used for the diagnosis of intracranial tumors due to of its high soft-tissue resolution and multi-parameter and multi-sequence technology; however, it cannot accurately identify certain tumor lesions with atypical morphological characteristics such as PCNSL and HGG (6, 7). Compared with MRI technology, which relies on morphological imaging, the 18F-fluorodeoxyglucose positron emission tomography computed tomography (18F-FDG-PET/CT) imaging technology is the most widely used functional imaging method in diagnosis used to evaluate tumor glucose metabolism (8). Currently, there have been few studies on 18F-FDG-PET/CT for the differential diagnosis of the aforementioned tumors, but there has been no concrete evidence. The purpose of this study is to conduct a meta-analysis to assess the value of 18F-FDG-PET/CT imaging for the differential diagnosis of PCNSL and HGG.

## Materials and methods

### Literature search

All available literature pertaining to the research question that was published before July 2022 was searched on the PubMed, EMBASE, Cochrane library, web of science, China National Knowledge Infrastructure (CNKI), Wanfang, and China Science and Technology Journal (CQVIP) databases. The search terms: “lymphoma,” “gliomas,” and “positron emission tomography computed tomography” were used. Different Boolean logic retrieval methods are used for different databases. In order to search relevant literatures as comprehensively as possible, we used medical subject headings for retrieval. The key words are shown in Supplementary Table 1. In cases where the full text was unavailable; it was requested from the author through e-mail obtain as much as possible.

### Inclusion and exclusion criteria

The following inclusion criteria were used for the literature: (1) The subjects were PCNSL and HGG patients with a definite pathological diagnosis and PET/CT was utilized to distinguish these two tumors; (2) Literature with sufficient data to calculate the sensitivity (SEN) and specificity (SPE) and further calculate the true positive (TP), true negative (TN), false positive (FP), and false negative (FN); (3) The full-text review and final analysis are limited to articles published in Chinese and English. The exclusion criteria were as follows: (1) Duplicate publications; (2) Conference abstract, letter, case report, review, systematic review, meta-analysis, and other non-original literature publications; (3) Animal studies or *in vitro* research; (4) Articles with insufficient or unextractable data. In cases where the studies were by the same author or from the same cohort, the most recent studies with the largest sample size were included.

### Qualitative assessment of literature

The qualitative assessment of the included literature is especially important in a diagnostic meta-analysis, with the strict and accurate evaluation of the literature quality directly affecting the effectiveness and value of the results of the diagnostic meta-analysis. All the literature collected in this study was evaluated using the Quality Assessment Tool for Diagnostic Accuracy Studies (QUADAS-2) (9), revised by the Review Manager 5.3 software in 2011. The quality assessment was performed independently by two reviewers with a medical background and familiarity with diagnostic meta-analysis. Any discrepancy between the two reviewers was resolved through consensus or handed over a third reviewer for evaluation, if required.

### Data extraction

Two reviewers independently extracted the required data from all the selected literature articles and ensured that the included data adhered strictly to the requirements of the study and maintained consistency of the results. Any discrepancy in the data extraction was resolved through consensus or handed over to a third reviewer for evaluation, if needed. The extracted data included: first author, literature characteristics (year of publication, country, and region), research type (retrospective/prospective study), and patient characteristics (number of patients, age, percentages male and female). For each study, the equivalent of true positives (TP), true negatives (TN), false positives (FP), and false negatives (FN) was extracted, where TP represents the number of patients with a true diagnosis of PCNSL by PET-CT, TN represents the number of patients with a true diagnosis of HGG by PET-CT, TN represents the number of patients misdiagnosed with PSCLS by PET-CT, and FN represents the number of patients misdiagnosed with HGG by PET-CT. Sensitivity (SEN) was defined as TP/(TP + FN), and specificity (SPE) was defined as TN/(FP + TN). If the article did not provide it directly, the required data were obtained through the corresponding calculations. SEN represents the ability to correctly diagnose PCNSL, and SPE represents the ability to correctly diagnose HGG.

### Statistical analysis

Testing the heterogeneity of the included literature is an important step in meta-analysis. The heterogeneity of diagnostic meta-analysis is primarily attributable to the threshold effect or non-threshold effect using Meta-DiSc 1.4 (XI Cochrane Colloquium, Barcelona, Spain) (10). The commonly used method to determine the threshold effect is to assess the threshold effect between SEN and 1-SPE using the Spearman correlation coefficient. If the correlation coefficient P < 0.05, it indicates is the presence of the threshold effect. In case no heterogeneity is caused due to the threshold effect, the Cochrane Q test and I^2^ test is needed to evaluate and detect heterogeneity, which is caused by the non-threshold effect (11). In this meta-analysis, the value of P <0.05 or I^2^ >50% indicates heterogeneity. The bivariate effect model was used to combine statistics such as SEN, SPE, positive likelihood ratio (PLR), negative likelihood ratio (NLR), and diagnostic odds ratio (DOR) with 95% confidence intervals (CIs), plot summary receiver operating characteristic (SROC) curve, and to calculate the value of area under the curve (AUC). Fagan's nomogram was used to evaluate the a priori probability and a posteriori probability of PET / CT in distinguishing the two tumors (12). A sensitivity analysis was used to evaluate the stability of the results, and the Deek's test was used to judge the presence of bias in the study (13). The meta regression and subgroup analysis was used in order to further clarify the possible sources contributing to heterogeneity.

## Results

### Literature search

**Figure 1:**
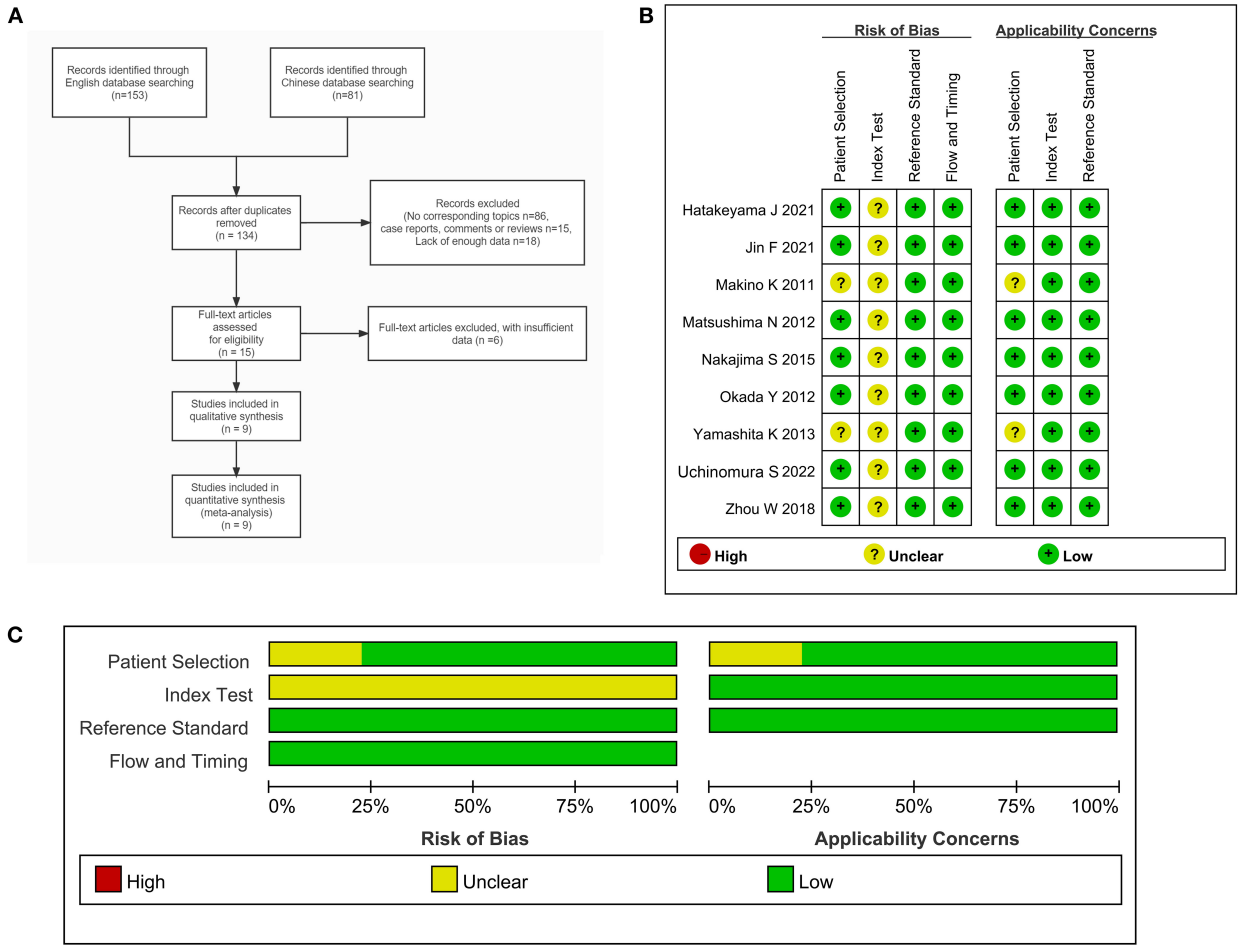
**(A)** Flow chart of the inclusion and exclusion of literature in this study. **(B)** Document quality evaluation chart. **(C)** Methodological qualitative analysis of the included studies.

In this study, three Chinese and four English language databases were searched according to the retrieval process provided by the Meta-analysis (Preferred Reporting Items for Systematic Reviews and Meta-Analyses, PRISMA) guidelines. Initially, a total of 234 literatures were searched, which included 153 English and 81 Chinese language literature. The Endnote X7 software was used to manage the literature obtained from seven databases, and a total of 100 duplicate literature items were removed, leaving 134 research items. A systematic search was conducted for the remaining articles, 86 articles were found to be inconsistent with the research question and were excluded after title and abstract screening. Fifteen non-original research items such as conference abstracts, letters, case reports, and reviews were excluded, while 18 articles were excluded due to insufficient data. Subsequently, the full texts of the 15 remaining literature articles were downloaded for a detailed screening. After excluding six literature articles that could not be used to extract the necessary data, and nine literature articles were finally included for the meta-analysis. The screening and inclusion process for the literature is shown in Figure 1A.

### Literature characteristics and quality assessment

A total of nine studies (14–22) were included in this study (Table 1), including one Chinese and eight English language literature articles. The included literature was published between 2011 and 2022, and contained a total of 151 PCNSL patients and 281 HGG patients, aged between 49 and 90 years. All the included studies were retrospective studies, and the patients were from China or Japan. The diagnosis of all the patients were confirmed using histopathology. After using the QUADAS-2 diagnostic quality tool for evaluation of the quality of the nine included literatures, it could be observed that relatively few of literature articles have an unclear risk for patient selection due to the lack of basic patient information. Further, as the cut-off values of all studies are not pre-specified, there is an unclear risk for coefficient evaluation. However, the reference standard, flow, and timing for the all included studies are low risk and low concern. In terms of the overall inclusion, the quality of the study was considered satisfactory (Figures 1B,C).

**Table 1:** Basic clinical characteristics and parameters of the included study.

**References**	**Year**	**Language**	**Country**	**Study design**	**PCNSL**			**HGG**			**Tp**	**Fp**	**Fn**	**Tn**	**Cut-off value**
					**No. of patients**	**Age**	**Sex (Male/Female)**	**No. of patients**	**Age**	**SEX (Male/Female)**					
Makino et al. (14)	2011	English	Japan	Retrospective	14	NA	NA	7	NA	NA	14	2	0	5	SUV_max_ of 12
Matsushima et al. (15)	2012	English	Japan	Retrospective	6	58.5 ± 22.4	3/3	11	55.2 ± 23.8	4/7	6	0	0	11	SUV ratio of 2.3
Okada et al. (16)	2012	English	Japan	Retrospective	7	69.4 ± 11.6	3/4	11	49.3 ± 14.7	7/4	6	1	1	10	SUV_max_ of 12
Yamashita et al. (18)	2013	English	Japan	Retrospective	19	64.8 ± 10.5	NA	37	58.5 ± 16.7	NA	18	8	1	29	SUV_max_ of 19
Nakajima et al. (17)	2015	English	Japan	Retrospective	11	70 (39–79)	4/7	23	56.5 (16–90)	13/10	11	5	0	18	SUV_max_ of 9.35
Zhou et al. (19)	2018	English	China	Retrospective	40	56.80 ± 9.44	25/15	52	57.29 ± 11.64	27/25	31	4	9	48	SUV_max_ of 13.77
Hatakeyama et al. (20)	2021	English	Japan	Retrospective	20	70 ± 8.7	9/11	55	66 ± 1.5	33/22	19	2	1	53	SUV ratio of 2.07
Jin et al. (21)	2021	Chinese	China	Retrospective	23	63.3 ± 9.5	11/12	21	60.9 ± 14.0	11/10	19	5	2	18	SUV_max_ of 12.7
Uchinomura et al. (22)	2022	English	Japan	Retrospective	13	70 (54–87)	11/2	62	70 (18–85)	30/32	9	4	4	58	SUV ratio of 2.65

SUV, standardized uptake value; NA, no answer.

### The results of meta-analysis

**Figure 2:**
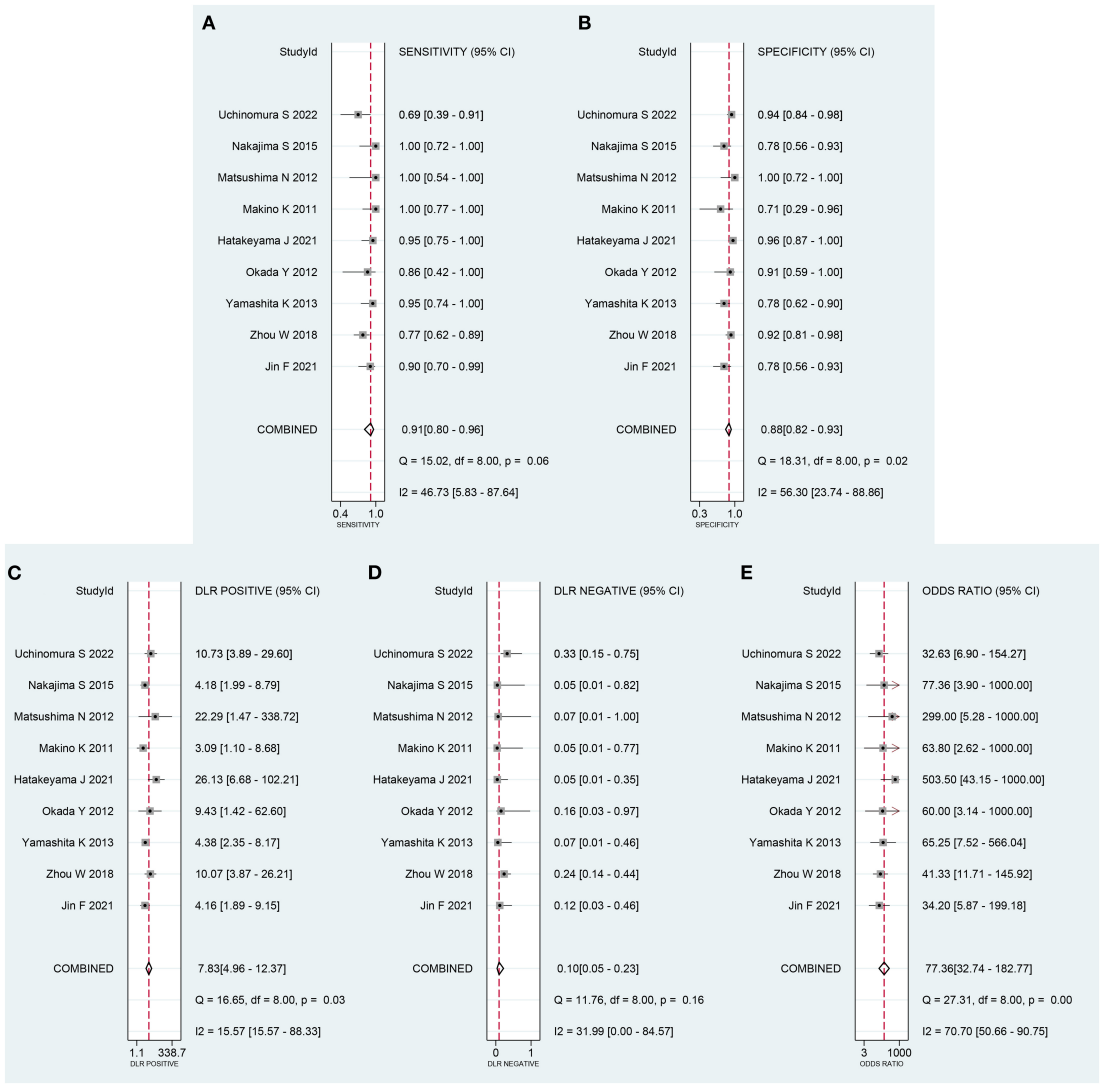
Forest plot of combined **(A)** sensitivity (SEN) and **(B)** specificity (SPE), **(C)** positive likelihood ratio (PLR), **(D)** negative likelihood ratio (NLR) and **(E)** diagnostic odds ratio (DOR).

**Figure 3:**
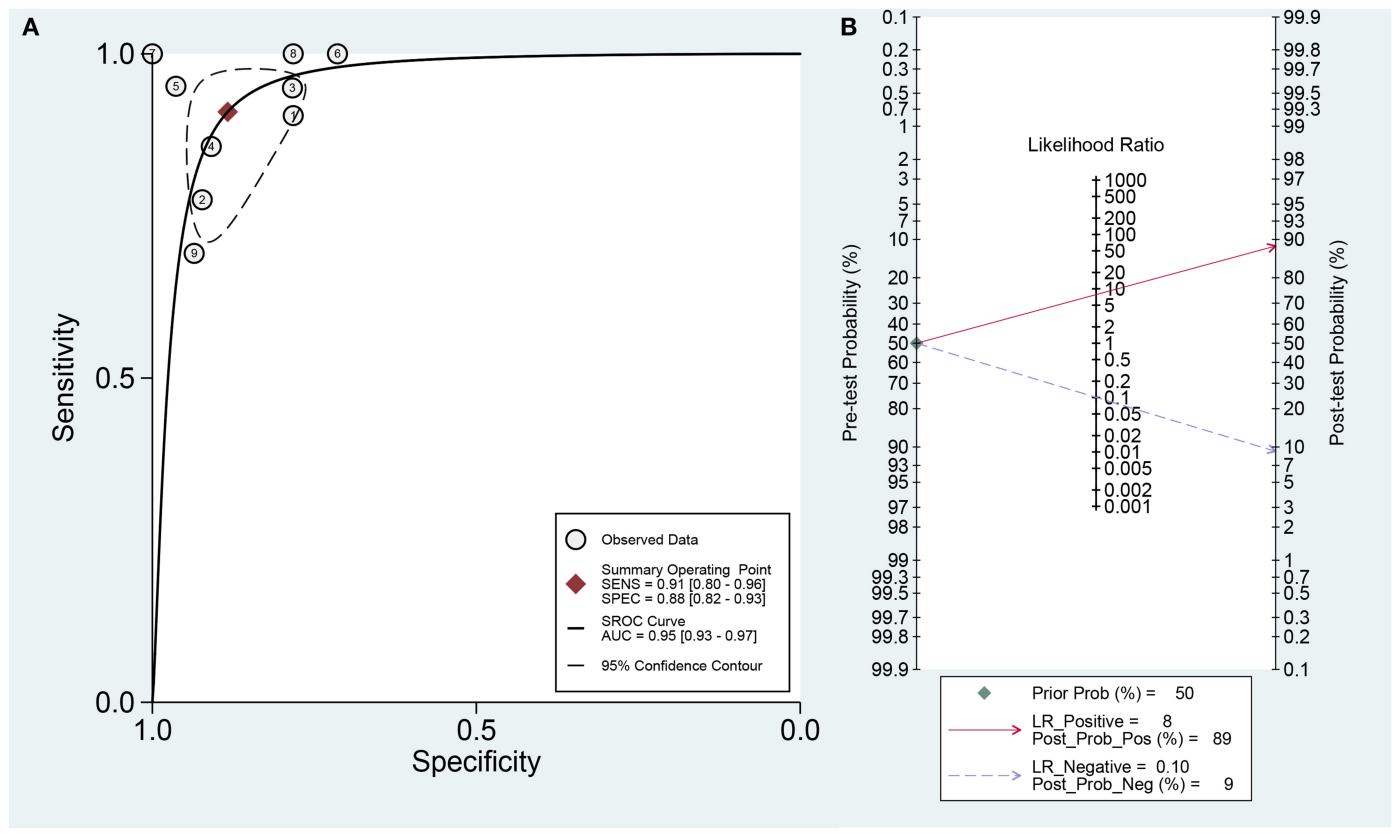
**(A)** The area of curve (AUC) of Summary receiver operating characteristic (SROC) curve. **(B)** Fagan's nomogram for assessing post-test probabilities of 18F-FDG-PET/CT.

The data was imported into the MetaDiSc 1.4 software for analysis, and the Spearman correlation coefficient between SEN and (1-SPE) is 0.450 and *P* = 0.224, which indicates the absence of a threshold in this study. The results of the heterogeneity test were: Q = 6.910, df = 2.00, *P* = 0.016, I^2^ = 71%, which suggests the presence of some heterogeneity in this study. The combined SEN was 0.91 (95% CI: 0.80–0.96; I^2^ = 46.73%), combined SPE was 0.88 (95% CI: 0.82–0.93; I^2^ = 56.30%), the combined PLR was 7.83 (95% CI: 4.96–12.37; I^2^ = 15.57%), combined NLR was 0.10 (95% CI: 0.05–0.23; I^2^ = 31.99%), and combined DOR was 77.36 (95% CI: 32.74–182.77; I^2^ = 70.70%) (Figures 2A–E). The AUC of SROC was 0.95 (95% CI 0.93–0.97) (Figure 3A). The analysis of Fagan nomogram shows the probability before prediction is 50%. In cases where the results of PET/CT are positive, the probability of diagnosing PCNSL will increase to 89%. In cases where the result is negative, the probability of diagnosing PCNSL will reduce to 9% after detection (Figure 3B).

### Sensitivity analysis and publication bias

**Figure 4:**
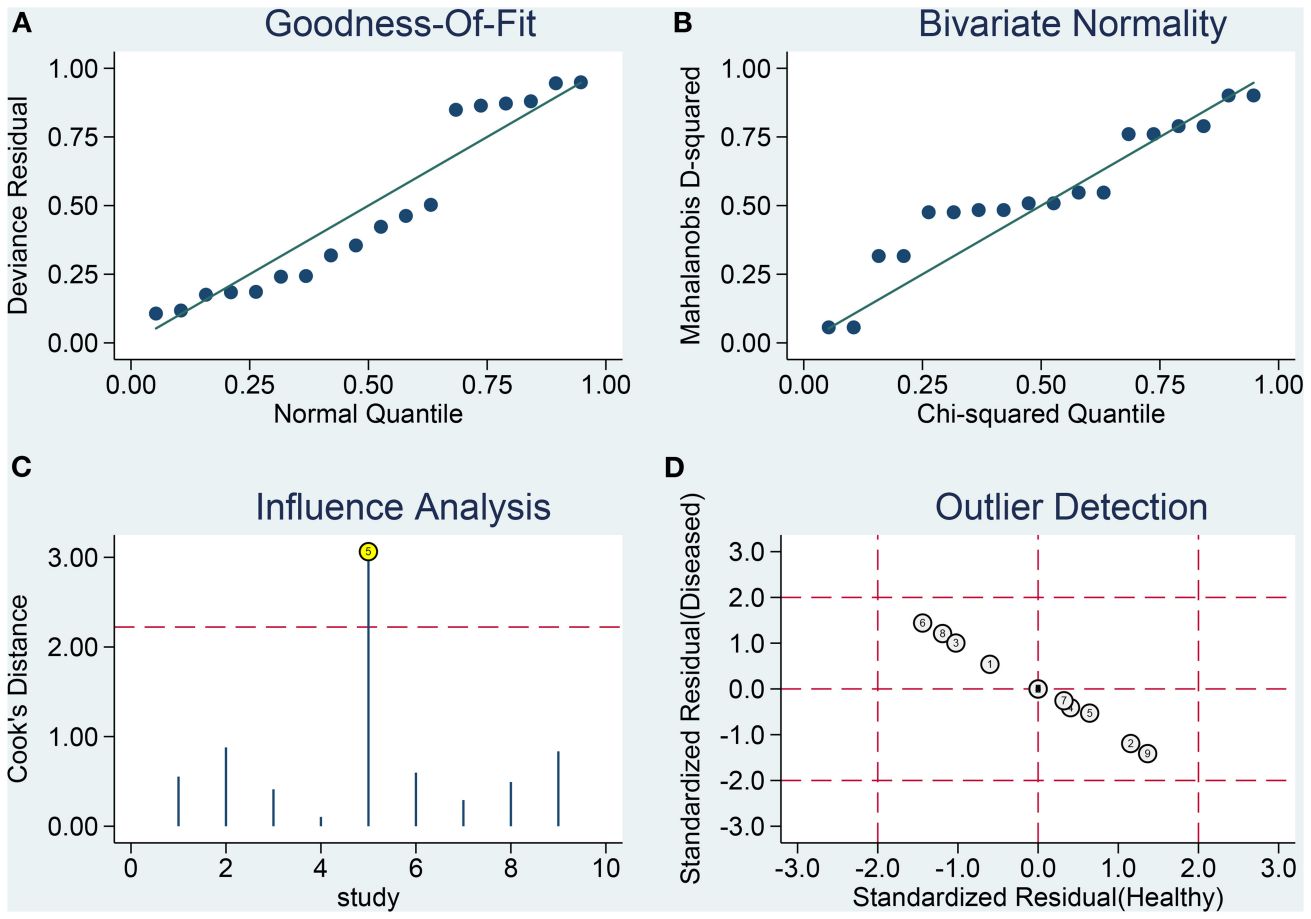
Influence analysis and outlier detection. **(A)** goodness-of-fit, **(B)** bivariate normality, **(C)** influence analysis, and **(D)** outlier detection.

**Figure 5:**
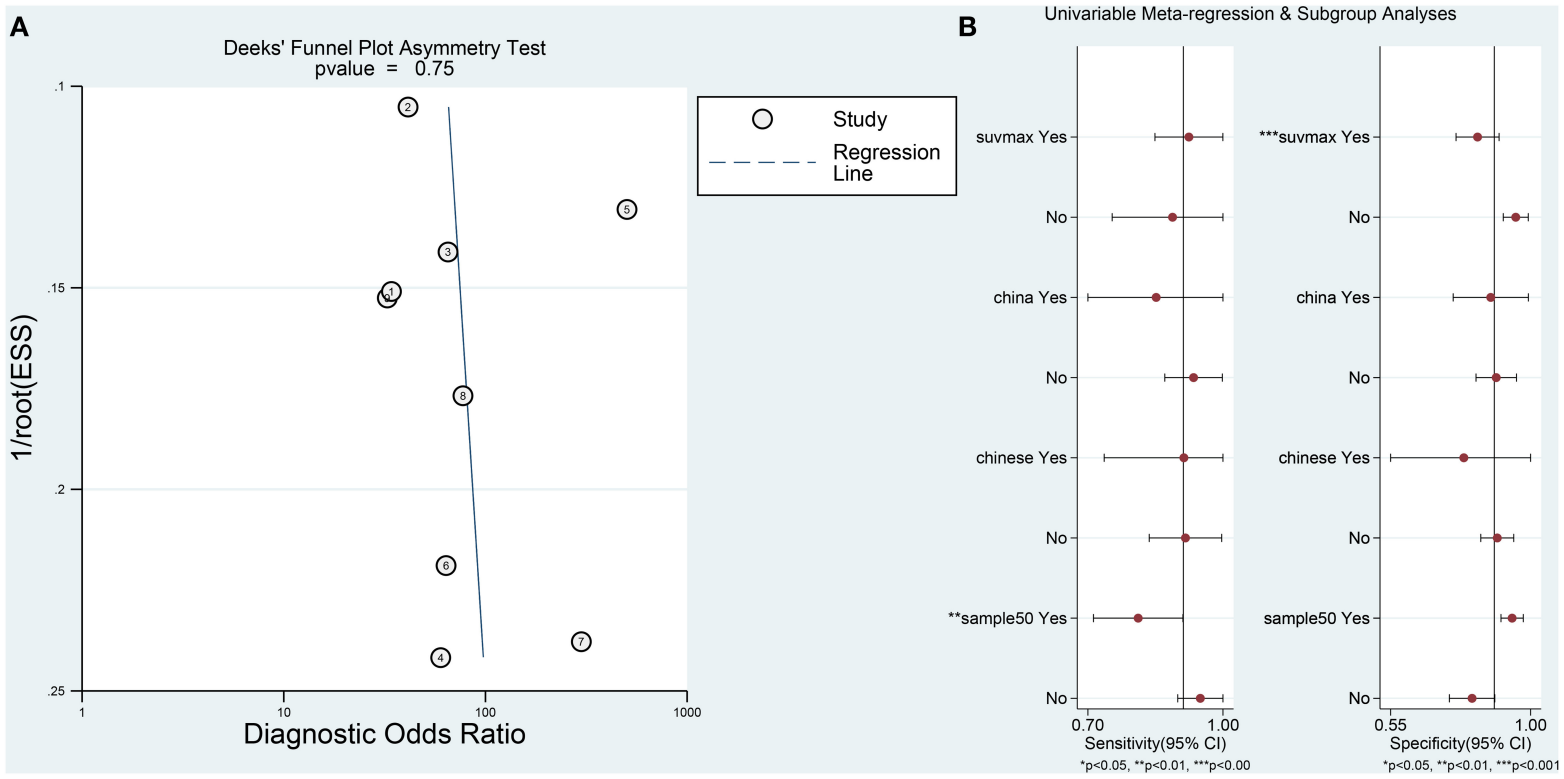
**(A)** The results of Deeks' funnel plot asymmetry test to assess publication bias. **(B)** Univariable meta-regression analysis for sensitivity and specificity of the 18F-FDG-PET/CT diagnosis of PCNSL and HGG.

Sensitivity analysis revealed that it was possible to conduct the goodness of fit and bivariate normality tests using the bivariate mixed-effect model for this meta-analysis (Figures 4A–D). One abnormal study was excluded and the combined SEN was 0.91 (95% CI: 0.79–0.96; I^2^ = 44.94%), combined SPE was 0.86 (95% CI: 0.79–0.92; I^2^ = 44.49%), the combined PLR was 6.61 (95% CI: 4.27–10.22; I^2^ = 0), combined NLR was 0.11 (95% CI: 0.05–0.25; I^2^ = 18.18%), and the combined DOR was 60.74 (95% CI: 25.26–146.05; I^2^ = 26.93%). The AUC of SROC was 0.94 (95% CI 0.91–0.96). Compared to the previous results, these results did not indicate a significant change. The Deek's publication bias test was conducted for all the nine selected studies, as shown in Figure 5A. The symmetrical funnel chart shows that there is no publication bias in this study (*P* = 0.69).

### Meta-regression and subgroup analysis

Furthermore, in order to determine additional likely sources of heterogeneity, a meta-regression analysis was conducted based on four indicators: sample size (≤ 50 vs. >50), language of the selected literature (Chinese vs. English), patient's country (Japan vs. China), and parameters (standardized uptake value (SUV) max vs. SUV ratio). As is shown in Table 2 and Figure 5B, the sample size was found to be the primary reasons for heterogeneity of combined sensitivity. For the results of merge specificity, different parameters are the main reason for heterogeneity.

**Table 2:** Results of meta-regression analysis and subgroup analysis.

**Covariate**		**No. of studies**	**Sensitivity [95%CI]**	**P1**	**Specificity [95%CI]**	**P2**	**χ^2^**	***P***
Sample_size	>50	3	0.81 [0.71–0.91]	0.87	0.83 [0.76–0.90]	<0.01	11.40	<0.01
	<50	6	0.95 [0.90–1.00]		0.81 [0.74–0.89]			
Language	Chinese	1	0.91 [0.73–1.00]	0.65	0.79 [0.55–1.00]	0.14	1.40	0.50
	English	8	0.92 [0.83–1.00]		0.89 [0.84–0.95]			
Country	China	2	0.85 [0.70–1.00]	0.13	0.87 [0.75–0.99]	0.19	2.38	0.30
	Japan	7	0.93 [0.87–1.00]		0.89 [0.83–0.95]			
Parameter	SUVmax	6	0.92 [0.85–1.00]	0.87	0.83 [0.76–0.90]	<0.01	7.41	0.02
	SUV ratio	3	0.89 [0.75–1.00]		0.95 [0.91–0.99]			

## Discussion

In this study, after an exhaustive search of Chinese and English language databases, based on stringent inclusion and exclusion criteria, nine relevant literature articles were selected for the meta-analysis. No publication bias was found in this meta-analysis, and the results indicated that 18F-FDG-PET / CT had a positive effect on the differential diagnosis of PCNSL and HGG. Additionally, sensitivity analysis demonstrated that the selected studies and results from the analyses were highly robust. However, it is worth noting that there is some heterogeneity in our meta-analysis. The heterogeneity of merger sensitivity and merger specificity, as determined using further meta-regression analysis and subgroup analysis, it is found to be attributable to the sample size and the country of the patient and the use of different parameters, respectively. In general, 18F-FDG-PET / CT has a good prospect in differentiating the above two tumors.

The PCNSL and HGG are intracranial tumors with a high degree of malignancy with an ever-increasing incidence rate. The aggressive growth rate exhibited by both the tumors result in a similar cell density. Even though differences in pathological manifestations and lesion distribution can be ascertained under the microscope, the two tumors are difficult to distinguish using imaging. Moreover, owing to the differences in their treatment methods, it is crucial to determine an accurate conclusion, through imaging, prior to operation. In addition to Computed Tomography (CT), the primary method for imaging of craniocerebral diseases is craniocerebral Magnetic Resonance Imaging (MRI), which has the ability to diagnose as well as differentially diagnose intracranial diseases (23–25). For example, a meta-analysis of 18 studies by Du et al. suggested that DWI could differentiate between PCNSL and HGG with an AUC of 0.90 (26). Another meta-analysis of 10 articles by You et al. suggested that arterial spin labeling could differentiate between PCNSL and HGG with an AUC of 0.86 (27). Additionally, a diagnostic model constructed with a deep learning algorithm based on MR imaging was used to differentiate PCNSLs, glioblastoma and brain metastases, with an AUC of 0.98 in the differential diagnosis of PCNSL (28). However, PET-CT still has great diagnostic advantages over other techniques in terms of imaging detection methods that rely on a single index.

PET / CT imaging is an advanced imaging technology that can simultaneously capture the cellular, molecular level images and anatomical structures through an image-fusion technology. At present, the most commonly used tracer is 18F-FDG. In recent years, the application of functional imaging (such as 18F-FDG-PET/CT) in the prognostic evaluation and diagnosis of PCNSL has gained momentum and gradually increased (29, 30). 18F-FDG PET / CT can reflect the anatomical and metabolic information of the structure under focus at the same time, which can significantly increase the diagnostic accuracy of PCNSL (31). Since PCNSL has a high level of tissue metabolism, enhanced anaerobic glycolysis, rapid proliferation, and limited interstitial components, PCNSL often shows an abnormal increase of FDG uptake, which is the most prominent difference in HGG using PET / CT imaging.

Two main parameters used in the study that are included in this meta-analysis, namely SUVmax and SUV ratio. As the most commonly used metabolic parameter of PET / CT, SUVmax is a semi-quantitative measure that relies on the degree of tumor metabolism, which reflects the metabolic activity of the tumor tissue when the uptake of 18F-FDG is at its highest. The tumor uptake of 18F-FDG depends on an increase in the number of functional glucose transporters and glycolytic enzymes in metabolically active cells and is positively correlated with the degree of malignancy of the tumor. Therefore, SUV can be used as an important semi-quantitative parameter to describe the characteristics of tumor metabolism. SUVmax in the PCNSL group was significantly higher than that of the HGG group, suggesting that metabolism of central nervous system lymphoma was higher than that of HGG (31). The high metabolism may be attributable to the high invasiveness of diffuse large B-cell lymphoma, which had an active value-added and high uptake of the metabolic imaging agent, 18F-FDG. Further, the current diagnostic cutoff values of SUVmax included in the study are set at nearly 9–19, which enables an accurate distinction between the two tumors. However, a study has reported a contradictory finding indicating that a value of SUVmax > 25 points to the presence of a lymphoma (32). Due to the frequent development of necrotic foci in malignant tumors, which may affect the average standard tumor uptake, and the near impossibility of excluding all necrotic areas in the process of delineating regions of interest, the SUV ratio has also been proposed for the differential diagnosis of PCNSL and HGG. In terms of the SUV ratio, this study is generally consistent with our view that the SUV ratio is >2.

There was some heterogeneity in this meta-analysis, which can be explained by a number of reasons. Sample size could explain the heterogeneity of the sensitivity values; a small sample size may lead to uncertainty in the hypothesis testing results, while a larger sample size may lead to greater confidence that the hypothesis testing results are correct. Language and country did not have heterogeneous effects on sensitivity and specificity. However, a recent meta-analysis identified language and country as possible sources of heterogeneity in the differential diagnosis of PNCSL and HGG using DWI. There is still some debate about which is the best differential diagnosis parameter between SUVmax and SUV ratio. Makino et al. (14) and Kosaka et al. (33) compared the activity uptake rate of SUVmax and the focal side/control side SUV ratio, and concluded that SUVmax was the optimal parameter for differentiating HGG from PCNSL, believing that the background metabolic heterogeneity of the brain parenchyma had no influence on the experimental results. Meric et al. (34) took the SUVmax ratio of the lesion side/control side as the optimal parameter for differential diagnosis, considering that SUVmax is affected by multiple factors such as blood glucose level, age, environment and emotion. All these need to be further confirmed by prospective studies with large samples in the future.

It is worth noting certain limitations of the current meta-analysis: (1) relatively to other studies, fewer literature articles have been included, and the included literature is comprised of retrospective studies, which may contain some bias that needs to be further confirmed using prospective, high-quality studies. (2) the research articles included in the literature belong to Japan and China; therefore, there is a lack of data from Europe, America, and other countries or regions, which affects the representativeness of meta-analysis to a certain extent. (3) Most studies are unclear on their use of the blind method, which may contribute to an interpretation bias. (4) The sample size of certain studies are small, which affects the quality of results for the combined literature analysis.

## Conclusion

In conclusion, the 18F-FDG-PET/CT imaging technology has a high accuracy for the differential diagnosis of PNCSL and HGG. Patients suspected to have either of the two kinds of tumors may potentially benefit from 18F-FDG-PET/CT imaging. A timely examination of the tumor also provides valuable reference and a solid basis to determine the course of clinical treatment and also holds a positive prospect for application. Further exploration of the prospective and standardized diagnostic scheme is necessary before it is widely adopted.

## Data availability statement

The original contributions presented in the study are included in the article/Supplementary material, further inquiries can be directed to the corresponding author.

## Author contributions

GZ, JL, and XH conceived and designed the study. GZ acquired, analyzed the data, and wrote the manuscript. XH reviewed the final manuscript. All authors read, approved the manuscript, and agree to be accountable for all aspects of the research in ensuring that the accuracy or integrity of any part of the work is appropriately investigated and resolved.

## Conflict of interest

The authors declare that the research was conducted in the absence of any commercial or financial relationships that could be construed as a potential conflict of interest.

## Publisher's note

All claims expressed in this article are solely those of the authors and do not necessarily represent those of their affiliated organizations, or those of the publisher, the editors and the reviewers. Any product that may be evaluated in this article, or claim that may be made by its manufacturer, is not guaranteed or endorsed by the publisher.
