# Supplementary material for: Use of 18F-FDG-PET/CT in differential diagnosis of primary central nervous system lymphoma and high-grade gliomas: A meta-analysis
Source: Front Neurol. 2022 Aug 17;13:935459. doi: 10.3389/fneur.2022.935459 (PMC9428250; doi:10.3389/fneur.2022.935459)
Supplement: Supplementary file 1 [file Data_Sheet_1.doc]

- PET-CT Scan
- PET-CT Scans
- Scan, PET-CT
- Scans, PET-CT
- PET CT Scan
- CT Scan, PET
- CT Scans, PET
- PET CT Scans
- Scan, PET CT
- Scans, PET CT
- CT PET
- Positron Emission Tomography-Computed Tomography
- PET-CT
- CT PET Scan
- CT PET Scans
- PET Scan, CT
- PET Scans, CT
- Scan, CT PET
- Scans, CT PET
- Lymphomas
- Sarcoma, Germinoblastic
- Germinoblastic Sarcoma
- Germinoblastic Sarcomas
- Sarcomas, Germinoblastic
- Reticulolymphosarcoma
- Reticulolymphosarcomas
- Germinoblastoma
- Germinoblastomas
- Lymphoma, Malignant
- Lymphomas, Malignant
- Malignant Lymphoma
- Malignant Lymphomas
- Gliomas
- Glial Cell Tumors
- Glial Cell Tumor
- Tumor, Glial Cell
- Tumors, Glial Cell
- Mixed Glioma
- Glioma, Mixed
- Gliomas, Mixed
- Mixed Gliomas
- Malignant Glioma
- Glioma, Malignant
- Gliomas, Malignant
- Malignant Gliomas
- Glioblastomas
- Astrocytoma, Grade IV
- Astrocytomas, Grade IV
- Grade IV Astrocytoma
- Grade IV Astrocytomas
- Glioblastoma Multiforme
- Giant Cell Glioblastoma
- Giant Cell Glioblastomas
- Glioblastoma, Giant Cell
- Glioblastomas, Giant Cell
